# Prescribing sustainability: should UN sustainable development goals be part of the medical, pharmacy, and biomedical education?

**DOI:** 10.3389/fmed.2024.1438636

**Published:** 2024-10-07

**Authors:** Grégoire Wieërs, Simon Absil, Isabelle Maystadt, Charles Nicaise, Pauline Modrie, François-Xavier Sibille, Ludovic Melly, Jean-Michel Dogné

**Affiliations:** ^1^Department of Medicine, University of Namur, Namur, Belgium; ^2^Unit of Research in Clinical Pharmacology and Toxicology (URPC), University of Namur, Namur, Belgium; ^3^Department of Internal Medicine, Clinique Saint-Pierre, Ottignies, Belgium; ^4^Namur Research Institute of Life Sciences (NARILIS), Namur, Belgium; ^5^Institut de Pathologie et de Génétique, Gosselies, Belgium; ^6^Unit of Research in Molecular Physiology (URPHYM), Namur, Belgium; ^7^Institute of Health and Society, UCLouvain, Ottignies, Belgium; ^8^CHU UCL Namur, UCLouvain, Namur, Belgium; ^9^UCLouvain and Geriatrics Department, Clinical Pharmacy and Pharmacoepidemiology Research Group, Louvain Drug Research Institute, CHU UCL Namur, Yvoir, Belgium; ^10^Department of Surgery, CHU UCL Namur, Yvoir, Belgium

**Keywords:** sustainable development goals, health, prescription, systems thinking, pedagogy

## Abstract

**Introduction:**

How to adapt the curriculum of medicine, pharmacy, and biomedical sciences to prepare future health professionals to meet the challenge of maintaining quality care in a period of socio-ecological crisis? Addressing connections between humanity and sustainable environment should include an analysis of the reciprocal influence of various ecosystems, since it is now clear that healthcare systems have an impact on ecosystems and vice versa. Here, we propose that integrating the United Nations Sustainable Development Goals (SDGs) into the curriculum could be a first step in such a transversal education.

**Methods:**

Members of the faculty of medicine at the University of Namur, Belgium, including teaching staff of the department of medicine, pharmacy, biomedical sciences and psychology, were invited to respond anonymously to a questionnaire about their views on the feasibility of integrating the SDGs into their teaching. A subsequent survey on students’ perceptions of such teaching was conducted by student representatives.

**Results:**

Seventy-nine percent of surveyed members of the medical faculty believe that it is possible to integrate SDGs into their lectures. However, 44–86% of them did not know how to integrate each individual goal. 94.4% of students would like SDGs to play a greater role in their education; 64.4% of them would integrate them into existing modules; 23.9% would create an optional module, and 11.9% would create a mandatory module.

**Conclusion:**

Sustainable Development Goals integration into the curriculum of medicine, pharmacy, and biomedical sciences is perceived as challenging in a dense teaching program. To clarify how SDGs can translate into traditional lectures, we provide for each SDG targeted applications for bachelor’s, master’s and continuing education.

## Introduction

Medical students around the world are aware of the importance of acquiring competences to face the socio-ecological challenges that lay ahead (1). Universities also recognize the need to actively integrate the dual notion of environmental impact on health, and the impact of healthcare systems on the environment (2). But how to implement specific teachings in medicine, pharmacy, psychology or biomedical sciences that address these challenges? On one hand, students are looking for active teachings that they can directly apply to their future careers (1, 3, 4). On the other hand, educationalists must manage many subjects in a limited amount of time, which leaves little time for new chapters related to ecological interactions that often seem far removed from their specialized practice or research.

The United Nations Sustainable Development Goals (SDGs) include 17 urgent calls for action by all countries in a global partnership; they were adopted by all United Nations Member States in 2015 as described on the UN website (Table 1) (5). SDGs do not offer direct guidance on health issues, except for SDG 3 “good health and well-being.” Nevertheless, a translation could be made between SDGs and teaching in medicine, pharmacy, psychology, or biomedical sciences. We will now review all SDGs and provide some examples of possible integration into traditional teaching modules (Table 2). The complexity of the SDGs lies in their interdependence, which is inherent to complex systems, at the heart of the System Dynamics model described in the Meadows Report (6). These studies confirm the need for a holistic healing practice based on a systemic thinking that was previously solely based on traditional knowledge (7).

**Table 1:** Survey results: academics and scientists of the faculty.

Could the content of your course be used to teach…	No		Yes, I already teach		Yes, I would teach	
SDG 1 No poverty	12	*44%*	11	*41%*	4	*15%*
SDG 2 Zero hunger	10	*48%*	5	*24%*	6	*28%*
SDG 3 Good health and well-being	9	*39%*	9	*39%*	5	*12%*
SDG 4 Quality education	14	*58%*	3	*13%*	7	*29%*
SDG 5 Gender equality	13	*62%*	5	*24%*	3	*14%*
SDG 6 Clean water and sanitation	15	*68%*	4	*18%*	3	*14%*
SDG 7 Affordable and clean energy	15	*71%*	2	*10%*	4	*19%*
SDG 8 Decent work and economic growth	17	*74%*	5	*22%*	1	*4%*
SDG 9 Industry, innovation and infrastructure	13	*54%*	5	*21%*	6	*24%*
SDG 10 Reduced inequalities	16	*76%*	2	*10%*	3	*14%*
SDG 11 Sustainable cities and communities	12	*57%*	4	*19%*	5	*24%*
SDG 12 Responsible consumption and production	13	*59%*	6	*27%*	3	*14%*
SDG 13 Climate action	18	*86%*	1	*5%*	2	*9%*
SDG 14 Life below water	15	*68%*	4	*18%*	3	*14%*
SDG 15 Life on land	12	*57%*	4	*19%*	5	*24%*
SDG 16 Peace, justice and strong institutions	13	*62%*	5	*24%*	3	*14%*
SDG 17 Partnerships for the goals	11	*52%*	5	*24%*	5	*24%*

**Table 2:** Ways to integrate the SDGs into bachelor’s, master’s and continuing education.

Health—environment associations and SDG	Bachelor lectures	Master/continuing education	Subjects	Open access reviews
Air quality *SDG 7, 9, 12, 14, 15*	Biochemistry cancer physiology, cellular physiology, genetics, and embryology	General practice, pneumology, cardiology, dermatology, oncology, and pediatrics	Oxidative denaturation, alteration of signaling and metabolic pathways, pro-oncogenic effects, co-benefit of air pollution reduction, developmental consequences	(41, 42)
	Respiratory physiology, immunology	Pneumology	Inflammatory phenomena triggered by irritation, bronchospasm and asthma, allergization, Co-benefits of using inhaled powders rather than puffs	(43, 44)
	Cardiac physiology	Cardiology	Oxidative stress, vasospasm, atherogenesis, coronary disease.	(45)
Halogenated gases *SDG 9,11, 12*	Cell physiology, physics, and chemistry	Dermatology, anesthesiology	Ozone layer, UV and mutagenesis, gaseous anesthetics, atmospheric persistence, greenhouse effect, recycling	(46)
Airborne and waterborne pathogens *SDG 1, 3, 6, 9, 14*	Microbiology	Infectiology	Emerging pathogens and contaminants, zoonoses, farm monitoring, One Health	(47)
	Epidemiology	Infection control	Spread of epidemics, preventive measures	(48)
	Embryology	Neonatology	Developmental defects related to emerging pathogens	(49)
	Biology and statistics	epidemiology	Distribution of risk factors, populations statistics, ecological statistics, cartographic modeling	(50)
Energy *SDG 8, 9, 12, 13*	Physics	Digital tech, radiology, and laboratory medicine	Energy dependency, medical equipment life cycle	(51, 52)
		Surgery, intensive care, and hospital management	Surgical set composition, carbon footprint of sterilization vs. single-use equipment, waste risk classification and disposal routes	(53)
Xenobiotics *SDG 4, 5, 6, 9, 10, 14, 15*	Biochemistry, endocrine physiology	Endocrinology, pediatrics, obstetrics, and reproductive health	Persistence of metabolites in the environment, biological effects	(54, 55)
	Pharmacology	Clinical Pharmacology	Drug metabolism, PBT assessment (Persistence, Bioaccumulation, Toxicity)	(56, 57)
	Renal physiology	Nephrology	Individualization of drug dosage, excretion and concentration of bioactive metabolites in urine. Drinking water monitoring.	(58)
	Biology, physics	Radiology, nuclear medicine	Management of effluents, iodine, radionuclides, energy consumption—rational use of examinations (CT-NMR-PET scanner)	(25)
	Psychology	Pediatrics, dermatology	Xenobiotic sources, developmental and behavioral origins and consequences	(59)
Antibiotic resistant bacteria *SDG 3, 4, 6, 17*	Microbiology	Infectiology	Antibiotic stewardship, alternative approaches to antibiotic therapy	(60)
	Gut physiology	Gastroenterology	Microbiota alterations	(61)
Heavy metals, microplastics, waste management *SDG 6, 9, 11, 12*	Biochemistry, cancer physiology, and endocrine physiology	Endocrinology, oncology, and rheumatology	Alteration of metabolic pathways	(62)
		Radiology, cardiology, and laboratory medicine	Conductive catheters, implanted equipment, consumables, sampling equipment (needles), catheters, tubes (fluor), management of liquid and solid laboratory effluents, cell cultures (fetal calf serum), and animal materials.	(63, 64)
		Hospital management	Criteria for awarding contracts, objectives	(65)
		Pharmacy	Impact of drug manufacturing processes, transport and packaging on the environment.	(66)
Alimentation *SDG 1, 2, 10, 12, 17*	Biochemistry	Nutrition and endocrinology	Lipid metabolism (plant–animal), origin of omega-3 (small fish/krill and overfishing), and in-patient nutrition (qualitative/quantitative).	(67)
			Animal/vegetable proteins	
	Physiology	Endocrinology	Metabolic implications of food composition	(68)
Systems interactions - *SDG 10, 11, 16, 17*	Statistics, biology	Internal medicine	Ecology and resilience.	(69)
			Decompartmentalization of knowledge.	
			Planetary limits.	
Extreme climate *SDG 1, 3, 4, 6, 11, 13*	Physiology	Internal medicine, pediatrics	Heat stroke, consequences on the presentation of acute and chronic pathologies.	(70)
			Effect of alterations.	
	Physiology	Emergency, neurology, and women health	Crisis management, adaptation of practices	(71–73)
Population movements *SDG 1, 2, 11, 16*	Ethics, psychology	International law, infectiology, psychiatry, and emergency	Refugee law, (non)-communicable diseases	(74)
Invasive species *SDG 10, 11, 13, 15*	Biology	Infectiology	Adaptation to living conditions.	(75)
			Modification of nosography	
	Microbiology		Emerging arthrozoonoses	
Wellbeing / Peace *SDG 3, 4, 5, 16, 17*	Psychology	Psychiatry	Eco-social determinants of health. Eco anxiety.	(76)
	Religious sciences, philosophy	Humanities, nutrition	The role of technology in healthcare.	(77)
			Joint project.	
			Dialogue, relationship, responsibility.	
			Quality/quantity of life.	
	Philosophy	Ethics	Autonomy, responsibility, Bioethics	(78)
Leadership *SDG 8, 13, 16, 17*	Introduction to medical practice, pharmacology	Medical ethics, relationship training	Care access, gender equity, socio-economic and environmental cost of care, transhumanism: repair and transform, environmental justice, populations movements, health of individuals, and health of communities.	(74, 79, 80)

SDG 15, “Life on Land,” refers to the need for protection of land-based ecosystems. Exercising in natural settings benefits both mental and physical health by reducing the risk of cardiac diseases and obesity while enhancing immune functions and alleviating anxiety, fatigue, and depression (7, 8). Promoting nature-based social prescription also fosters outdoor exercise, social contact and integration with natural processes (9). Such observations could be echoed in almost all physiology and clinical lectures such as cardiology, pneumology, geriatrics, general surgery, orthopedics, internal medicine, neurology, psychology, and psychiatry.

SDG 2 “Zero Hunger” refers to the onset of diseases due to malnutrition. This could be included not only in clinical lectures in physiology, gastroenterology, and pediatrics, but also in fundamental biochemistry courses by integrating the impact of malnutrition on cellular metabolism. In this context, malnutrition should also be associated with nutritional disparities in the Global North and Global South and could integrate The Lancet EAT concepts, a rationale for diet adaptation integrating nutritional needs, food sources availability and planetary bounds (10).

SDG 13 “Climate Action” could be related to the geographic spread of disease vectors such as mosquitoes or ticks, due to global warming and could therefore be included in epidemiology or infectiology lectures (11–13).

The recent pandemics of SARS-CoV2 have revealed that actions needed to positively impact the health of individuals involve global initiatives that require communication skills to collaborate with non-medical disciplines such as economics, law, logistics or politics by developing cross-disciplinary educational units to achieve SDG 17 “Partnerships For the Goals” and SDG 1 “No Poverty.”

Reaching SDG 11 “Sustainable Cities and Communities” could impact the prevention and management of pathologies associated with insufficient practice of outdoor activities and overeating. This includes obesity, joint tenderness, depression, and social isolation and could be addressed in endocrinology, physiotherapy, geriatrics, and bariatric surgery lectures (11, 14, 15).

SDG 10 “Reduced Inequalities” could be introduced with the sources of health threats as excessive heat forcing populations of the Global South to migrate, or in the Global North as socio-economic determinants of health as housing conditions exacerbated by poverty e.g., humid living spaces, contamination by mold, or intense electromagnetic radiation (16). Exposure to pollution affects men and women differently, particularly in the context of fertility and metabolism. SDG 5 “Gender Equality” could thus be translated into the identification of environmental risk factors affecting men and women differently, such as the effect of diethylstilbestrol on the development of external genitalia and fertility; factors associated with the prevention of obesity or biliary lithiasis, physiology and endocrinology; insulin resistance in the context of exercising, influence of endocrine disruptors found in food and drinking water, nutrition; high glycemic index carbohydrate and accompanying substances in junk food, or ethics as the meeting of sex-related specific needs to achieve equity or egality.

Even more intriguingly, we observe that healthcare professionals could contribute to environmental change understood as the transformations of the ecosystem due to the overstepping of planetary limits (16, 17). Far from being limited to SDG 7 “Affordable and Clean Energy” and carbon dioxide emissions, healthcare infrastructures emit specific pollutants: xenobiotics, bacteria, viruses, gases, and radioisotopes. Carbon dioxide emissions by healthcare settings is associated with the production of other pollutions. Both are related to the prescription of pharmaceutical products that should be produced, transported, delivered and once absorbed by patients, re-emitted in the form of xenobiotics. Awarding contracts based on the industry’s commitment to environment preservation and on the internal policy of prescribing non-pharmacological approaches concerns management and leadership of healthcare settings as well as physicians and pharmacists (18–20). Pollution could relate to SDG 14 “Life Below Water” and SDG 6 “Clean Water and Sanitation.” This is especially relevant in contexts concerning sexual health such as oral contraception and hormone-replacement therapy, and exposure to endocrine disruptors in drinking water. These issues are of interest to the fields of gynecology, urology and endocrinology especially when considering that all possible hormone based treatment do not have the same persistence, bioaccumulation or toxicity once reemitted in the environment (21). In this regard, one can notice that today’s practice of medicine, a reflection of Western modernity based on reasoning, science and consumerism, may also contribute to social change in loss of caring for others, to the detriment of SDG 3 that specifically addresses health. In this context, indigenous conceptions could usefully enrich our modern approach to human health and the health of the planet. Indigenous Peoples ‘tend to approach health as an equilibrium of spirituality, traditional medicine, biodiversity and the interconnectedness of all that exists. This leads to an understanding of humanity in a significantly different manner that non-Indigenous Peoples’ (22). Mother Earth’s health and Indigenous Peoples’ health are synonymous.

SDG 9 “Industry, Innovation and Infrastructure” echoes that pollution associated with caregiving is almost unavoidable but could be reduced by wastewater treatment systems and carbon neutral energy production. Additionally, the overuse of medical services should be addressed to reach SDG 12 “Responsible Consumption and Production” to reduce both pollutions, iatrogenic diseases and public spending on healthcare. Inappropriate medical prescription negatively impacts health, economics and ecosystems. Globally, the overuse of medical services, defined as the provision of medical services for which the potential for harm exceeds the potential for benefit, is estimated between 30 and 80% of all medical services around the world. It affects both the Global North and the Global South and coexists with unmet medical needs: the so-called underuse. Both overuse and underuse highlights the need for teaching students to identify the adequacy of prescription for each individual as well as the duration of treatment (23, 24). In diagnostics, for example, physicians in the Global North quickly resort to the use of CT scans for screening purposes or positron emission tomography scans outside of formal indications, with the corollary not only of unnecessary exposure to radiations but also of overdiagnosis (25). Overuse also occurs in the context of inappropriate prescription of therapeutic means as antibiotics that contribute to accelerated emergence of antibiotic-resistant microbes (26, 27). Such observations raise the question of SDG 4 “Quality Education” to improve the rationale of prescription and to promote disease prevention (28). Van Rensselaer Potter, oncologist at the University of Wisconsin-Madison, was the first physician to use the neologism “bioethics” to describe the wisdom required to use the techniques and knowledge of life sciences to ensure the survival of humankind in the face of damage caused to the natural and social environment on which it depends (29–31). We could also examine on how medicine, pharmaceutical molecules or research contribute to conferring to each individual a “Decent Work” (SDG 8) and “Peace and Strong Institutions” (SDG 16) as an effect of efficient healthcare services with appropriate prescriptions and technical examinations using molecules and material produced by industries respectful of the well-being of their workers and of the environment and reduced dependency to non-renewable resources. In this respect, it is interesting to note that Schumacher College in the United Kingdom (Devon), known for its “Head-Heart-Hands” approach to transformative and holistic education, combines the acquisition of knowledge with the development of wisdom (32). The concept of ‘transformative education’ is also used by the United Nations Educational, Scientific and Cultural Organization (33). This innovative approach to education is part of the new social contract on education called for in a report on the future of education (34).

**Figure 1:**
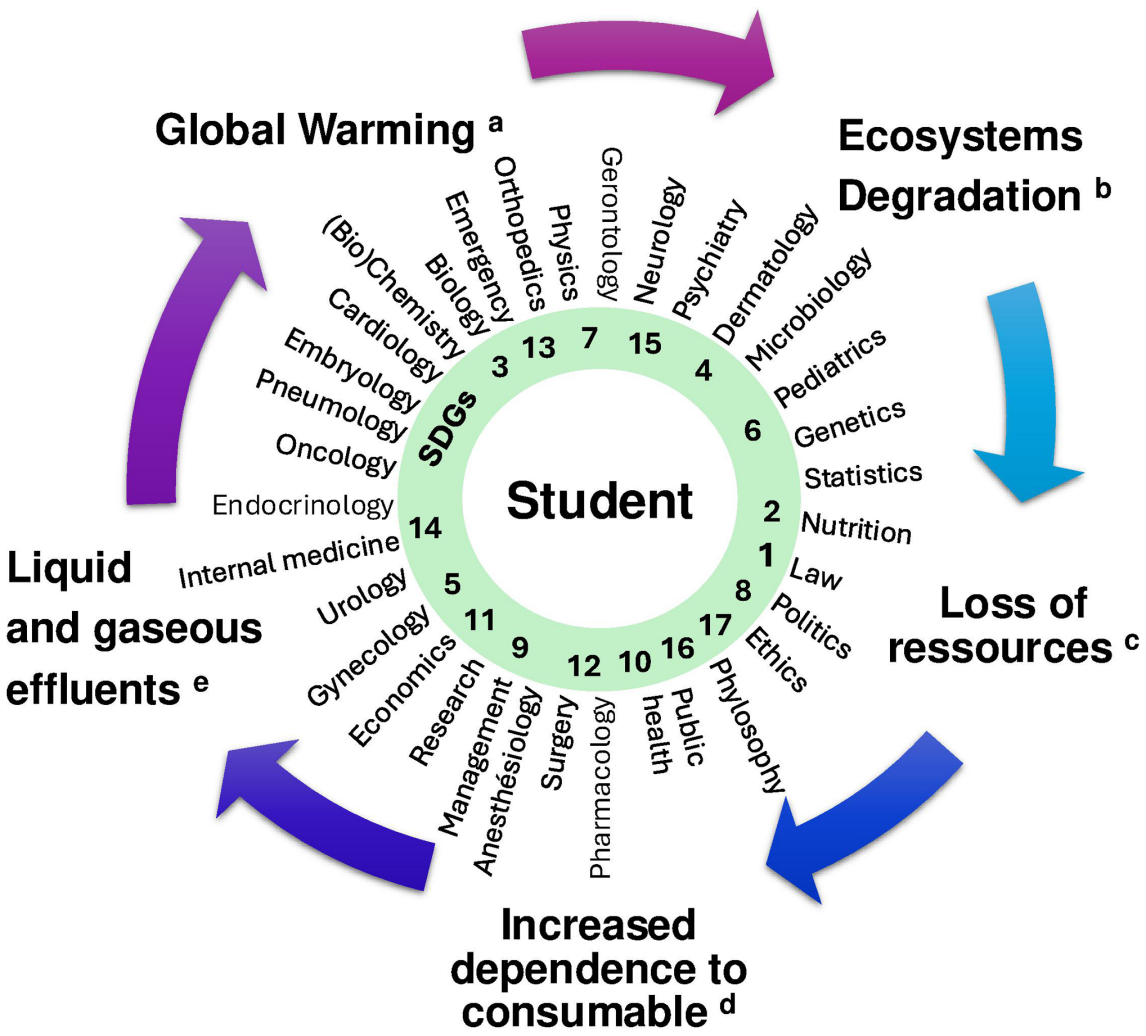
How the SDGs can be implemented in classical lectures. The Faculty Perspective showing how the SDGs can be implemented in classical lectures and how they relate to environmental change, such as (a) global warming and the increased frequency of extreme events responsible for traumatic injuries and psychological stress, (b) how humans come closer to wild animals when ecosystems are degraded, which favors zoonoses and epidemics, but also how loss of contact with nature can reduce well-being, (c) how overeating or starvation could affect the normal development of children or induce epigenic changes, but also how food contaminated with xenobiotics could be related to diseases such as obesity and endocrine diseases or cancer, (d) but also how overtreatment can reduce lifespan, and (e) increase the production of xenobiotics and CO2, which in turn could affect the environment, the climate and indirectly the patients and public health. SDGs are numbered in the green ring, examples of applications in classical lectures are provided. (1) No poverty: interconnections between laws and medical resources distribution, (2) Zero hunger: undernutrition and overnutrition related diseases and strategies to prevent them, (3) Good health and well being: climatic and ecologic conditions related with physiological stresses and pathogens selection, (4) Quality education: science based medicine and fight against factoids, (5) Gender equality: sex-related effects of exposition to degraded environment, (6) Clean water and sanitation: waterborne diseases and epidemics related to pathogens and endocrine disruptors, (7) Affordable clean energy: energy production process and energy dependency of medical devices and drugs production, (8) Decent work and economic growth: medical innovation including consideration for energy dependency and effluents, (9) Industry, innovation and infrastructure: availability, efficacy, and efficiency of medication and hospital management toward a reduction of effluents emission, (10) Reduced inequalities: socio economical basis of health and diseases, (11) Sustainable cities and communities: vaccination, reduction of antibiotics use, (12) Responsible consumption and production: rationale for prescription and deprescription, (13) Climate action: prevention of traumatic diseases and stresses due to global warming, (14) Life below water: Persistence, Bioaccumulation, Toxicity of effluents and wastewater sanitation, (15) Life on land: outdoor activity, nature prescription, ecoanxiety, neurological development, (16) Peace, justice and strong institutions: systemic thinking, extreme event, and epidemics management, and (17) Partnerships for the goals: World Health Organization priorities and diseases prevention.

Therefore, we, as both professional healthcare providers and teachers, are convinced that integrating SDGs into medical education is crucial for training future healthcare professionals who will be qualified to address global health challenges and who will promote sustainable practices in their communities. Figure 1 and Table 2 summarize the links between medical school courses, the SDGs and interactions with the environment.

## Methods

We conducted a pre-implementation survey in the form of multiple-choice questionnaires using the survey web service Drag’n survey (Créteil, France). This questionnaire was developed to identify isolated initiatives and interest in implementing concepts related to SDGs in faculty members. Faculty members received information about on SDGs and their potential implementation in lectures during a plenary presentation. Thereafter, an invitation to take part to the survey was sent by internal mail to 137 individuals engaged in teaching at the faculty of medicine at the University of Namur, Belgium, including 87 belonging to the academic body (63 men and 24 women) and 50 belonging to the scientific body (22 men and 28 women). The faculty of medicine comprises four departments: Pharmaceutical Sciences, teaching to pharmacy students, Medical Sciences teaching to medicine students, Psychology involved in various teachings, and Biomedical Sciences teaching to biomedical scientists.

The questionnaire was carried out by the director of the department, which conducts research on the theme of sustainable development in healthcare in the Center for Bioethics of the University of Namur (35, 36). The Dean of the faculty reviewed questionnaire. The first three questions focused on the perceived role of the practices taught in environmental change. Question 1: According to your experience and knowledge based on facts: “The practice of medicine, biomedical research, prescription and effects of pharmaceutical molecules (finish the sentence, tick all applicable boxes). Answers proposed: (A) changes due to environmental alterations, (B) leads to changes in the environment that harm ecosystems, (C) leads to changes in the environment that harm other humans, (D) does not have to involve concepts relating to the environment, and (E) no opinion.

Question 2: As a professional involved in population health, could you indicate your level of acceptance of the following statement by moving the cursor further to the right than you agree with the following: “Humanity has a responsibility to maintain a viable environment for all: humans and non-humans. The practice of research, pharmaceutical sciences and medicine must therefore include care for the environment in their prerogatives.” Participants can choose between 5 levels of agreement ranging from 0 disagree to 4 strongly agree.

Question 3: Could the content of your lecture contribute to the teaching of SDGs in biomedical sciences, pharmaceutical sciences or medical sciences? Answers proposed: (1) no, (2) yes, I am already teaching SDGs, and (3) yes, I would like to teach SDGs.

The next 17 questions (Annex 1) were created using a stereotypical format: each of the SDG’s title was translated into French, followed by a brief example of how it applies to the curriculum topics. A link to the SDGs website was also included (5). Answers proposed: (1) no, this SDG cannot be integrated into my course, (2) yes, I am already teaching about this SDG, (3) yes, I would like to teach about this SDG. The respondents could skip some questions explaining why the total number of answers of each line can differ. For each question, the respondent can leave a comment and give up anonymity to be contacted again and be implicated in the project of implementing SDGs in lectures.

The results of the survey were presented to student representatives and to the academic staff during the first quarter of 2023–2024 academic year. Student representatives elected by classmates: five men and three women with a mean age of 21.5 years old (age range 18–36 years old), were invited to reflect on how SDGs could be implemented in their curriculum. Consequently, second year undergraduate students interested in implementing SDGs in their curriculum, spontaneously carried out a survey among their 142 classmates with two questions: “Would you like sustainable development to have a more important role in your curriculum?” The second question focused on the mode of implementation: integration into existing courses, creation of an optional module, or creation of a mandatory module. Respondents were given the option of leaving anonymous comments.

The responses were automatically collected by the survey system and analyzed using basic descriptive statistics. The comments were summarized using two Artificial Intelligences: Gemini (Google AI) and Chatbot GPT (Open AI) in their free versions. The summaries were then refined by the author and included in the results.

## Results

### Results from the teaching staff survey

A total of 29 people answered at least one of the questions on a voluntary and anonymous basis. Fifteen were from the department of medical sciences, eight from the department of biomedical sciences, and six from the department of pharmaceutical sciences.

According to their experience and knowledge, 79% of the respondents thought that their profession and the effects of pharmaceutical molecules lead to changes in the environment that harm ecosystems; 59% thought that their profession and the effects of pharmaceutical molecules change due to environmental alterations; and 58% of the respondents thought that their profession and the effects of pharmaceutical molecules lead to changes in the environment that harm other humans. None of the respondents thought that their profession and the effects of pharmaceutical molecules do not have to involve concepts relating to the environment; 8% had no opinion.

Seventy percent of the respondents (14/20) strongly agreed with the following statement: “Humanity has a responsibility to maintain a viable environment for all: humans and non-humans. Therefore, the practice of research, pharmaceutical sciences and medicine must include environment preservation in their prerogatives.” Ten percent of the respondents chose one of the three intermediate agreements.

Only six out of 28 members of the faculty of medicine (21%) considered it impossible to integrate SDGs into their courses. When asked about each of the SDGs, between 44 and 86% of respondents thought that this specific SDG could not be implemented in their lectures. Their comments during the plenary presentation highlighted a lack of understanding of the relevance of these SDGs in the topics covered in their lectures. The results for questions 4–20 are summarized in Table 1.

Fifty open-ended comments were left by 10 faculty members. Five were between 40 and 50 years old, and three over 50 years old. Five men and three women gave up anonymity to be included in the subject development process. These comments revealed a commitment by faculty members to integrate the SDGs into their teaching, although still in a partial way and often limited to specific projects undertaken by student-faculty tandems. Issues such as “low-tech” solutions and access to medicines in low-income countries are occasionally discussed, but not consistently developed. The most frequently mentioned issues are the social determinants of health, ethical issues, the environmental impact of medical practice and research, and links to the One Health approach, particularly for colleagues also lecturing in the veterinary department. However, several barriers are mentioned. Firstly, the lack of time: already overloaded programs leave little room for new content. Secondly, the lack of resources: faculty members express the need for specific training to integrate the SDGs into their teaching. Thirdly, the disciplinary focus: medical education is often highly specialized, which can limit a more global and interdisciplinary approach. Faculty members also express an interest in further developing these themes further, in particular by broadening the scope of student projects so that they address issues related to the SDGs, presenting the results of these projects to a wider audience to raise awareness among students and colleagues, and integrating the SDGs more systematically into mainstream courses.

### Results from the undergraduate student survey

Of the 142 students, 51 men and 91women aged 18–36 years old (mean age 20.2 ± 2.2 years) enrolled in the second year of the Bachelor of Medicine program, 71 answered the question “Would you like to see sustainable development given more prominence in your courses?” 94.4% answered in the affirmative. Of these, 64% would like to see SDGs integrated into existing modules, 23% would like to see it as an optional module, and 13% would like to see it as a mandatory module. They were given the opportunity to leave an anonymous comment. The summary of their requests concerns, on one hand, the course content, which should enable them to better understand their role as healthcare professionals in sustainable development. On the other hand, they would like the SDGs to be viewed not as an end goal, but as a means of raising awareness about broader global issues beyond individual patient health. Students would like to see active pedagogy and coherence between SDGs and the university’s commitment to reducing the use of disposable materials and investing in the renovation of its buildings, for instance.

## Discussion

The aim of this study was to find an appropriate methodology to implement the SDGs in the curriculum of students of the Faculty of Medicine. Twenty-nine faculty members, corresponding to 21% of the included people, responded to the survey designed to identify initiatives in line with this project and the degree of feasibility; half of them are members of the medicine faculty. This proportion of respondents exceeds the proportion of 11.64% of people suffering eco anxiety in the general population of French speaking people in Europe and Africa (37). Most respondents (79%) believed their professions and pharmaceutical molecules can impact the environment, which is more than the 59% who thought environmental changes influence their professions and the effects of pharmaceutical molecules highlighting the perception of a responsibility in adapting teachings to climate change.

Only 21% of faculty members find it impossible to integrating SDGs into their courses. However, understanding of the relevance of these SDGs within their teaching was minimal. Academics highlighted the difficulty in translating the broad and interdisciplinary nature of the SDGs into specific subject areas within the medical curriculum. This underscores the need for innovative approaches to embed these goals in a way that resonates with various disciplines. In response to this need, we propose several examples that could facilitate SDGs integration into healthcare teaching in Table 2 and Figure 1, to promote both interdisciplinary understanding and practical application. Such an input remains partial and should be considered rather as a pedagogical implementation of systemic thinking into specialized education by linking physiopathological phenomena with environmental and social point of action to prevent emergence of diseases or inequity.

On the other hand, 50% of students answered the survey conducted by the student representative, a twofold difference with the people implicated in teaching in the faculty of medicine. Although the survey methodology was different, the participation to the survey is consistent with the proportion of younger people concerned by climate change in the general population. These students emphasized the necessity of integrating SDGs into the curriculum, not merely as an additional topic but as a transformative element that enhances the practice of their future profession. They advocate a pedagogical shift from a competitive, subject-based approach to a more collaborative, practice-oriented educational model. This shift could transform healthcare provision, fostering greater emphasis on prevention and non-pharmacological approaches to disease management. By promoting interprofessional collaboration among doctors, pharmacists, and researchers, this educational model is in line with the interdisciplinary nature of the SDGs. It also meets the educational commitments of the University of Namur, whose goal is to educate competent and responsible citizens to serve a multicultural society based on each individual personal experience in the Ignatian Pedagogical Paradigm (38).

The results of our survey suffers several biases. First, the use of multiple-choice questionnaires with oriented solutions may produce influenced responses. Second, the questionnaire was not formally validated and was initially intended to gather preliminary opinions from colleagues and students on the implementation of lectures that incorporate the role of the natural environment in health and disease. Third, the small sample size of both faculty members and bachelor students and the northwestern cultural homogeneity are other constrains on the generalizability of these observations. Despite these limitations, the survey reveals a significant interest in SDGs among Belgian French-speaking academics and students and identifies specific challenges related to their integration into medical education.

Although SDGs are inherently interdisciplinary, they do not explicitly address health topics, except the SDG 3. Contrary to industrial processes that could be directly concerned by SDGs, health sciences seem to be in the need of a supplementary interpretation of how to implement each of the SDGs. In Table 2 and Figure 1, we offer examples of how to create links between SDGs and health topics. Such a strategy allows a significant degree of pedagogical flexibility. This flexibility can be harnessed to develop innovative teaching strategies that emphasize the interconnectedness between health and environmental sustainability. For instance, integrating case studies that explore the impact of environmental and social factors on health can help students appreciate the relevance of ecological considerations in clinical practice. Following the example of the implementation of the SDGs in other curricula, medical education should integrate systems thinking and create a link between the teaching of life sciences and other disciplines that can have an impact on the environmental parameters necessary for health. For example, teaching green chemistry, which integrates the SDGs, can contribute to reducing environmental pollution and favors a healthy aging (39). Such teaching requires collaboration between the disciplines of health and chemistry or other industrial processes. In the pedagogical integration of systems thinking in chemistry education as in medicine visual summaries linking input, output, and global consequences of activities as system-oriented concept map extension (SOCME) represent easily implementable strategies in classical lectures (40). Graphical representations could be adapted to medical education by including both intended and unintended changes to the planetary cycle associated with activities in health care settings, which in turn could impact public or individual health as we propose in Figure 1 for the medical curriculum.

## Conclusion

Our findings indicate a significant interest and a recognized need among both students and academics for integrating SDGs into medical education. Addressing the challenges identified and harnessing the interdisciplinary nature of the SDGs can play a pivotal role in preparing future healthcare professionals to practice medicine, pharmacy, and biomedical in ways that are both ecologically responsible and socially relevant. This integration can drive a broader transformation in healthcare, aligning it more closely with the principles of sustainability and global health equity. Our experience in implementing SDGs into the curriculum of our medical school underscores the value of an active pedagogy that allows student participation in program adaptation. It also highlights the complexity faced by educators in creating meaningful connections between subjects and SDGs.

The integration of SDGs into medical education is not only feasible: it is mandatory. By embedding these goals within the curriculum, we can ensure that future healthcare professionals possess the knowledge and skills necessary to respond to ecologically driven health events and contribute to sustainable development. Our role as a university is to train citizens capable of taking action to meet the challenges of their era. This approach will enhance the relevance and scope of healthcare education, cultivating a generation of professionals committed to advancing global sustainability goals.

## Data Availability

The raw data supporting the conclusions of this article will be made available by the authors, without undue reservation.
