# Supplementary material for: Prescribing sustainability: should UN sustainable development goals be part of the medical, pharmacy, and biomedical education?
Source: Front Med (Lausanne). 2024 Oct 7;11:1438636. doi: 10.3389/fmed.2024.1438636 (PMC11492205; doi:10.3389/fmed.2024.1438636)
Supplement: Supplementary file 1 [file Table_1.DOCX]

Annex 1. Questionnaire

ODD1: No Poverty

Does your lecture cover the socio-geographical determinants of disease, the identification of poverty criteria or the impact of natural disasters and on health or socio-economic repercussions? The impact of poverty on the preservation of ecosystems? Low-tech research? Poor countries' access to pharmaceutical molecules? Biomedical/medical research in low-GDP countries?

ODD2: Zero Hunger

Do you address in your lecture the causes and consequences of deficiencies and their link with agriculture or veterinary science / by including them in a reflection inspired by the “one health” project? The need for healthy ecosystems for food production?

ODD3: Good health and Well-Being

Does your lecture cover the causes and consequences of health crises, the states and functions that enable “well-being”, disease prevention, notions of health economics or evidence-based public policy?

ODD4: Quality Education

Do you address in your lecture the links between cultural, sporting, social and economic development and health? Health risks linked to communication (artificial intelligence, social networks)? Ideologies of change? Access to training in research, pharmaceutical sciences, biomedical sciences or medical sciences? Inter-university exchanges?

ODD5: Gender Equality.

In your lecture, do you discuss how health crises or physical determinants (sexual dimorphism, hormonal influences, metabolism) can lead to gender inequality?

ODD6: Clean Water and Sanitation

Does your lecture cover pathogens and diseases linked to unsafe water? Xenobiotics, drug metabolites and effluents linked to medical activity, research or pharmacological applications? Sanitation of drinking water?

ODD7: Affordable and Clean Energy

Does your lecture address the dependence of the pharmaceutical industry, medical technology and research on energy production? Technologies to reduce this dependence? Diseases caused by combustion-related fine particles? The greenhouse effect and its link with healthcare practice/research?

ODD8: Decent Work and Economic Growth

Do you address the influences of working conditions on health in your course? Respectful relationships at work, cooperation with other professions, interest in non-medical disciplines such as law or economics that influence healthcare practice?

ODD9: Industry, Innovation and Infrastructure

In your lecture, do you question the place of innovation in the health sciences in the perspective of sustainable development? The adaptation of medical / research / disposables logistics infrastructures to crises? The influence of research, care and health industry on ecosystems?

ODD10: Reduced Inequalities.

Does your lecture cover notions of access to information and technology? Determinants of access to healthcare? Promoting research in low-GDP countries?

ODD11: Sustainable Cities and Communities

Does your lecture deal with disease prevention / pharmacological resource requirements? The One Health approach? The impact of climate migration?

ODD12: Responsible Consumption and Production

Does your lecture cover the causes and consequences of medical / pharmacological over-consumption? Sustainable food and health?

ODD13: Climate Action

Does your lecture cover the choice of tests and therapies according to their environmental impact? How biomedical research can contribute to the fight against global warming?

ODD14: Life Below Water

Does your lecture cover the structure of drugs and their persistence in the environment? Water purification? Cumulative toxicity for aquatic ecosystems?

ODD15: Life on Land

Does your lecture cover notions of ecology or interdependence? Invasive species and emerging diseases? Genetic manipulation and mutagens? Pollution generated by health industry ?

ODD16: Peace, Justice and Strong Institutions

Does your lecture address notions of ethics and responsibility? Spirituality and links between economics, ecology, justice and health?

ODD17: Partnerships for the Goals

Does your lecture cover emerging diseases and demographics? WHO roles and recommendations? The purpose of medical research: the fight against malaria, tuberculosis, diseases linked to unsafe water, maternal and infant mortality, etc.? A perception of the human being as part of a wider ecosystem? Global access to treatments?
